# Associations of BMI with all-cause mortality in normoglycemia, impaired fasting glucose and type 2 diabetes mellitus among an elderly Chinese population: a cohort study

**DOI:** 10.1186/s12877-022-03382-z

**Published:** 2022-08-21

**Authors:** Rui Song, Xuejiao Chen, Kun He, Xueqi Hu, Kaizhi Bai, Wenlong Shi, Songhe Shi

**Affiliations:** Department of Epidemiology and Health Statistics, College of Public Health, Zhengzhou University, 100 Kexue Avenue, Zhengzhou, Henan 450001 People’s Republic of China

**Keywords:** Body mass index, Fasting plasma glucose, Type 2 diabetes mellitus, Mortality, Elderly population

## Abstract

**Aim:**

To explore the associations of body mass index (BMI) and mortality among people with normal fasting glucose (NFG), impaired fasting glucose (IFG), and type 2 diabetes mellitus (T2DM) in an elderly Chinese population.

**Methods:**

A retrospective cohort study was conducted that included 59,874 elderly people who were aged 60 and older at baseline. Data for the study came from a health check-up program in China between 2011 and 2019. Hazard ratios (HRs) and 95% confidence intervals (CIs) were calculated using multivariable Cox proportional hazard models of BMI categories by glycemic status.

**Results:**

During the median of 5.96 years of follow-up, 7928 participants died (6457/49057 with NFG, 712/5898 with IFG and 759/4919 with T2DM). In adjusted Cox models, risk of mortality showed a decreasing trend with BMI < 18.5 kg/m^2^, 24 ≤ BMI < 28 kg/m^2^, and BMI ≥ 28 kg/m^2^ compared to 18.5 ≤ BMI < 24 kg/m^2^: HR (95% CI): 1.33 (1.18 to 1.49), 0.88 (0.83 to 0.93), and 0.90 (0.82 to 0.98), respectively, for NFG; 0.89 (0.55 to 1.46), 0.84 (0.71 to 0.99), and 0.88 (0.70 to 1.11), respectively, for IFG; and 1.42 (0.88 to 2.29), 0.75 (0.64 to 0.89), and 0.76 (0.62 to 0.93), respectively, for T2DM. There were curvilinear-shaped associations between BMI and mortality in the NFG and T2DM groups (*P* overall < 0.001 and *P* overall < 0.001, respectively; *P* nonlinearity < 0.001 and *P* nonlinearity = 0.027, respectively) and no significantly association between BMI and all-cause mortality was observed in the IFG group (*P* overall = 0.170).

**Conclusion:**

High BMI compared to normal BMI was associated with decreased mortality, especially in the old populations with NFG and T2DM. Future studies are needed to explain the obesity paradox in elderly patients with T2DM.

## Background

An analysis of the Global Burden of Disease Study in 195 countries and territories observed that the number of global deaths and disability-adjusted life years (DALYs) attributable to high body mass index (BMI) substantially increased between 1990 and 2017. Successful population-wide initiatives targeting high BMI may mitigate the burden of a wide range of diseases [1]. According to an analysis of multiple cause of death data from both countries, overweight and obesity may be key drivers of the recent slowdown or reversal of the decline in cardiovascular death rates in Australia and the US [2].

In the general population, the association between obesity and increased risk of cardiovascular disease (CVD) has been well established [3, 4]. However, once CVD occurs, obesity paradoxically seems to confer a survival advantage. There is growing evidence that overweight patients with CVD survive longer than their normal weight counterparts, an effect called the obesity paradox [5]. Low BMI is predictive of increased mortality; this could potentially be mediated through known associations with weight loss, chronic diseases, frailty, and cachexia [6, 7]. Excess weight may result in metabolic reserves that protect against adverse outcomes. Although obesity is a major risk factor for type 2 diabetes mellitus (T2DM), underweight is associated with increased mortality compared to normal weight, and moderately elevated BMI is associated with decreased mortality. A similar obesity paradox may also exist after the development of T2DM.

Some studies have found that overweight or obese diabetic patients do not have a lower mortality rate at diagnosis than normal-weight diabetic patients or obesity paradox patients [8]. For instance, an AusDiab cohort study of 10,575 Australian adults aged 25–91 found no evidence of an obesity paradox among diabetic patients and no differences in the association between obesity (BMI ≥ 30 kg/m^2^) and mortality among participants with and without diabetes [9]. Some studies have found an obesity paradox, in which being overweight or obese reduces the risk of all-cause mortality in diabetic patients [10, 11]**.** A pooled analysis of 5 longitudinal cohort studies found that adults (age ≥ 65 years) who were normal weight (BMI, 18.5 < BMI ≤ 24.99 kg/m^2^) at the time of incident diabetes had higher mortality than adults who were overweight or obese (BMI ≥ 25 kg/m^2^) (HR, 2.23; 95% CI, 1.55 to 3.20) [12].

In summary, the results of such studies are not always consistent. The association between BMI and the risk of all-cause mortality has been studied in both patients with T2DM and elderly people, while similar studies are lacking in elderly patients with T2DM. Meanwhile, there may be different associations between BMI and mortality in elderly individuals with different glycemic statuses, which needs to be examined. Therefore, we examined the relationships between BMI and all-cause mortality at three fasting glucose levels in an elderly population. The hypothesis is that high BMI compared to normal BMI is associated with decreased risk of all-cause mortality at three fasting glucose levels among elderly people.

## Methods

### Study population

**Fig. 1:**
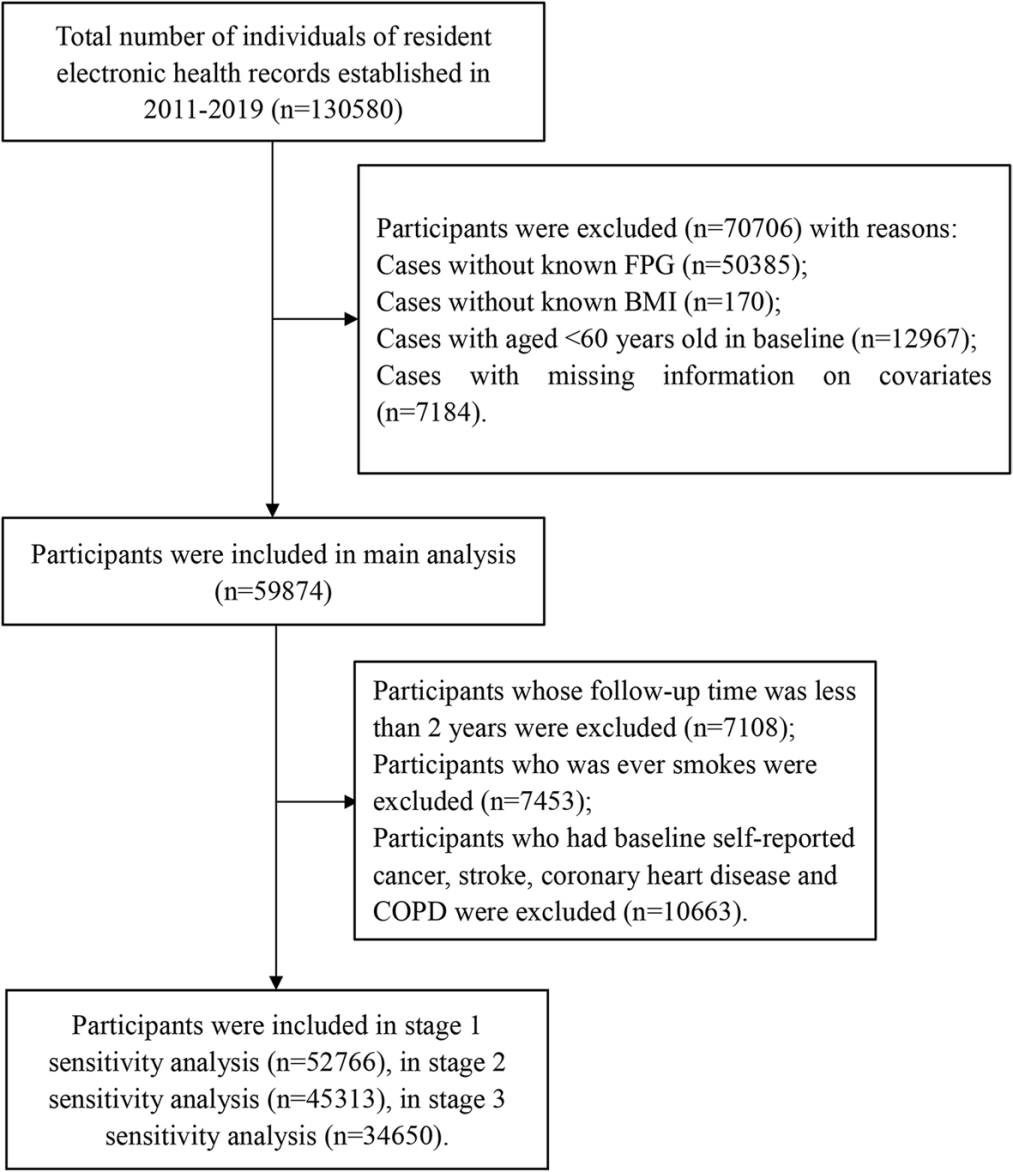
Flow chart of the study population

To monitor risk factors for all-cause death in elderly people and improve the health of urban and rural residents, an annual health check-up project has been carried out since 2010 in Xin zheng, Henan Province for the city’s elderly population aged 60 years and above [13]. We used data from the electronic health check-up database from January 2011 to November 2019. A total of 130,580 people entered the cohort. We excluded participants who had any of the following conditions: (1) missing information on fasting blood-glucose (*n* = 50,385); (2) missing information on BMI (*n* = 170); (3) age of under 60 years (*n* = 12,967); and (4) missing information on covariates (*n* = 7184). Ultimately, 59,874 participants were included in the analysis. A flowchart of the study participant selection process is shown in Fig. 1.

This was a retrospective population-based cohort study approved by the Ethics Committee of Zhengzhou University in China, and informed consent was obtained from each participant before data collection. Study procedures were performed in accordance with the Declaration of Helsinki ethical principles for medical research involving human subjects.

### Assessment of BMI and FPG

Height and weight were measured twice by trained nurses following rigorous protocols, and the average of each value was used for analysis. Body height of participants was measured without shoes using a stadiometer, and body weight was measured with participants in light clothing and without shoes using electronic scales [14]. BMI was calculated as weight (kg) divided by the squared height (m) and categorized into four groups according to Chinese standard of obesity: underweight (BMI < 18.5 kg/m^2^), normal weight (18.5 ≤ BMI < 24 kg/m^2^), overweight (24 ≤ BMI < 28 kg/m^2^), and obese (BMI ≥ 28 kg/m^2^) [15].

After fasting for at least 8 h, blood samples were collected from the participants. Fasting plasma glucose (FPG), total cholesterol (TC), and triglyceride (TG), were detected with an automatic biochemical analyzer (DIRUI CS380, Changchun, China) [16]. Participants were asked “Have you ever been diagnosed with or treated for diabetes by a doctor or other health professional?” FPG was used to classify glycemic status as NFG (< 6.1 mmol/L), IFG (≥ 6.1 and < 7.0 mmol/L) and T2DM (≥ 7.0 mmol/L) [17]. T2DM was defined as having FPG ≥ 7.0 mmol/L or using insulin or oral hypoglycemic agents, and/or as presenting a self-reported history of diabetes [18].

### Data collection

Other relevant data from the study included study participants’ demographic characteristics, lifestyle behaviors and clinical data. Demographic variables included age, sex (men/women), comorbidities (coronary heart disease (CHD), chronic obstructive pulmonary disease (COPD), cancer and stroke) and medical history of T2DM. Diagnosis of diseases was conducted according to the International Classification of Diseases. CHD included ICD-10 codes I20–I25, stroke included ICD-10 codes I60–I69, cancer included ICD-10 codes C00-C97, and COPD included ICD-10 code J44 [19]. Stroke was defined as sudden onset of a focal, nonconvulsive neurological deficit lasting more than 24 h. Lifestyle behaviors included smoking status (current smokers, former smokers, and never smokers) [20], alcohol consumption (never, once in a while, and more than once a week and every day), and physical exercise status (never, once in a while, and more than once a week and every day). Clinical data, including systolic blood pressure (SBP), TC and TG were measured by trained personnel.

### Outcome definition

The study cohort was observed until the end of 2019. The outcome of interest in this study was all-cause mortality. Death certificates were collected from the annual standard health check-up data with a digital linkage to the hospital dataset for admissions. Mortality data were obtained from the National Causes of Death Register.

### Statistical analysis

Participants’ baseline sociodemographic characteristics, clinical characteristics, and anthropometric measurements based on the three groups were presented. Descriptive data are presented as numbers (percentages) and quantitative variables are presented as medians (interquartile ranges (IQRs)) with median (IQR). Categorical data were analyzed using the chi-squared test. Continuous data were compared using the Kruskal–Wallis test.

First, we calculated the 9-year all-cause mortality by both glycemic categories and BMI categories. Then, we used Cox proportional hazard models to calculate the adjusted HRs with 95% CIs for the four BMI categories and the all-cause mortality for the three glycemic categories. With normal weight (18.5 ≤ BMI < 24 kg/m^2^) as the reference group, we constructed two Cox regression models: Model 1 was unadjusted, and Model 2 was adjusted for age, sex, smoking, alcohol consumption, physical activity, history of CHD, COPD, cancer and stroke, SBP and TC at baseline, with the proportional hazard assumption being satisfied. Linear trend tests were conducted by entering the median value of each category of BMI as a continuous variable in the models.

We also used restricted cubic splines to characterize the dose response association and explore the potential linear or nonlinear relationship between mortality and BMI on a continuous scale, with BMI 21 kg/m^2^ as the reference. The knots were placed at the 5th, 25th, 75th and 95th percentiles. The test result for overall association was checked first. If the test for overall association was significant, the test result for nonlinearity and linearity were checked, and the *P*-value for non-linear association < 0.05 indicated a significant result indicating the non-linear association.

For subgroup analyses, we stratified participants by age and sex at baseline. Finally, we performed a series of sensitivity analyses to test the robustness of our primary outcome, excluding (1) deaths within the first 2 years of follow-up; (2) ever smokers; and (3) participants who self-reported cancer, stroke, coronary heart disease and/or COPD at baseline.

Restricted cubic splines were performed in R × 64 4.0.4 (R Foundation for Statistical Computing, Vienna, Austria), and the forest plot was performed by Microsoft Excel 2010. The other analyses were performed by SPSS software, V.21.0 (SPSS, Chicago, Illinois, USA). A two-sided *P* value < 0.05 was considered to be statistically significant.

## Results

### Basic characteristics

At baseline, the median (IQR) age was 65 (62–72) years. Of the 59,874 participants, 49,057 (81.9%) had NFG, 5898 (9.9%) had IFG and 4919 (8.2%) had T2DM (Table 1). Elderly patients with diabetes had the highest levels of alcohol consumption. The levels of systolic blood pressure, triglycerides, total cholesterol and BMI increased as blood glucose levels increased (Table 1). Overweight and obesity accounted for 47.1% (NFG), 58.0% (IFG) and 62.7% (T2DM), respectively. The prevalence of self-reported pre-existing diseases, including CHD, COPD, cancer, and stroke, was highest in the T2DM group.

**Table 1:** Baseline characteristics of the included participants according to the level of FPG

	Total	NFG	IFG	T2DM	
Variable	*N* = 59,874	*N* = 49,057 (81.9)	*N* = 5898 (9.9)	*N* = 4919 (8.2)	*P* values
Age, years	65 (62–72)	65 (62–72)	65 (62–72)	66 (62–72)	0.001
SBP, mmHg	130 (120–145)	130 (120–142)	135 (121–150)	138 (125–150)	< 0.001
TG, mmol/L	1.26 (0.92–1.63)	1.23 (0.90–1.60)	1.34 (1.00–1.83)	1.48 (1.09–2.13)	< 0.001
TC, mmol/L	4.76 (4.18–5.36)	4.71 (4.16–5.31)	4.86 (4.23–5.53)	4.97 (4.31–5.62)	< 0.001
BMI, kg/m^2^	23.94 (22.09–26.35)	23.81 (21.99–26.08)	24.73 (22.76–27.21)	25.11 (22.99–27.55)	< 0.001
Sex					< 0.001
Male	28,367 (47.4)	23,654 (48.2)	2587 (43.9)	2126 (43.2)	
Female	31,507 (52.6)	25,403 (51.8)	3311 (56.1)	2793 (56.8)	
Smoking					< 0.001
Never	51,437 (85.9)	41,973 (85.6)	5155 (87.4)	4309 (87.6)	
Former	1300 (2.2)	1043 (2.1)	142 (2.4)	115 (2.3)	
Current	7137 (11.9)	6041 (12.3)	601 (10.2)	495 (10.1)	
Alcohol consumption					0.001
Never	55,915 (93.4)	45,863 (93.5)	5506 (93.4)	4546 (92.4)	
Once in a while	2365 (3.9)	1938 (4.0)	218 (3.7)	209 (4.2)	
More than once a week	504 (0.8)	416 (0.8)	50 (0.8)	38 (0.8)	
Every day	1090 (1.8)	840 (1.7)	124 (2.1)	126 (2.6)	
Physical exercise					< 0.001
Never	45,161 (75.4)	37,447 (76.3)	4246 (72.0)	3468 (70.5)	
Once in a while	3008 (5.0)	2514 (5.1)	265 (4.5)	229 (4.7)	
More than once a week	2013 (3.4)	1647 (3.4)	200 (3.4)	166 (3.4)	
Every day	9692 (16.2)	7449 (15.2)	1187 (20.1)	1056 (21.5)	
BMI (kg/m^2^) categories					< 0.001
BMI < 18.5	1288 (2.2)	1152 (2.3)	91 (1.5)	45 (0.9)	
18.5 ≤ BMI < 24	28,982 (48.4)	24,798 (50.5)	2390 (40.5)	1794 (36.5)	
24 ≤ BMI < 28	21,615 (36.1)	17,261 (35.2)	2305 (39.1)	2049 (41.7)	
BMI ≥ 28	7989 (13.3)	5846 (11.9)	1112(18.9)	1031 (21.0)	
Self-reported comorbidity					< 0.001
CHD, COPD, cancer, stroke	14,888 (24.9)	11,830 (24.1)	1597 (27.1)	1461 (29.7)	
Death	7928 (13.2)	6457 (13.2)	712 (12.1)	759 (15.4)	< 0.001

*SBP* Systolic blood pressure, *CHD* Coronary heart disease, *COPD* Chronic obstructive pulmonary disease

### BMI and all-cause mortality for each FPG level

The median follow-up time was 5.96 years (IQR: 3.50–7.49). The all-cause mortality in the underweight and normal weight ranges was significantly higher in patients with diabetes than in the NFG or IFG groups (Table 2). Among diabetic patients, mortality was highest at BMI < 18.5 kg/m^2^ and then decreased as the BMI category increased, with similar trends in the NFG and IFG groups. With BMI < 18.5 kg/m^2^, 18.5 ≤ BMI < 24 kg/m^2^, 24 ≤ BMI < 28 kg/m^2^, and BMI ≥ 28 kg/m^2^, the death rates (per 1000 person-years) were 79.46, 38.19, 24.88, and 25.04, respectively, for those with T2DM; 36.15, 28.37, 19.07, and 17.79, respectively, for those with IFG; and 48.38, 28.46, 19.82, and 18.48, respectively, for those with NFG (Table 2).

**Table 2:** BMI and all-cause mortality for each FPG level

Variables	BMI < 18.5	18.5 ≤ BMI < 24	24 ≤ BMI < 28	BMI ≥ 28	*P* for trend	Increase per SD^b^
NFG						
Deaths	291	3795	1811	560		
Death rate^a^	48.38	28.46	19.82	18.48		
Model 1	1.71 (1.52–1.93)	1.00 (reference)	0.70 (0.66–0.74)	0.65 (0.59–0.71)	< 0.001	0.78 (0.76–0.80)
Model 2	1.33 (1.18–1.49)	1.00 (reference)	0.88 (0.83–0.93)	0.90 (0.82–0.98)	< 0.001	0.92 (0.89–0.94)
IFG						
Deaths	17	364	231	100		
Death rate^a^	36.15	28.37	19.07	17.79		
Model 1	1.31 (0.81–2.13)	1.00 (reference)	0.68 (0.58–0.80)	0.65 (0.52–0.81)	< 0.001	0.81 (0.75–0.88)
Model 2	0.89 (0.55–1.46)	1.00 (reference)	0.84 (0.71–0.99)	0.88 (0.70–1.11)	0.169	0.96 (0.89–1.04)
T2DM						
Deaths	18	355	256	130		
Death rate^a^	79.46	38.19	24.88	25.04		
Model 1	2.11 (1.31–3.38)	1.00 (reference)	0.66 (0.56–0.78)	0.67 (0.54–0.81)	< 0.001	0.79 (0.73–0.85)
Model 2	1.42 (0.88–2.29)	1.00 (reference)	0.75 (0.64–0.89)	0.76 (0.62–0.93)	< 0.001	0.86 (0.80–0.93)

^a^Per 1000 person-years; ^b^Standard deviations for BMI: 3.2 kg/m^2^ in NFG group, 3.5 kg/m^2^ in IFG group, and 3.5 kg/m^2^ in T2DM group. Model 1 is unadjusted; Model 2 is adjusted for age, sex, smoking, alcohol consumption, physical activity, history of CHD, COPD, cancer and stroke, SBP and TC at baseline

Table 2 also presents the results from the Cox regression that estimated the association between the levels of BMI and all-cause death. The multivariable adjusted HRs (95% CI) per SD increase in BMI were 0.92 (0.89 to 0.94), 0.96 (0.89 to 1.04), and 0.86 (0.80 to 0.93) for NFG, IFG and T2DM, respectively. In the NFG and T2DM groups, the HRs for all-cause death decreased significantly with increasing BMI categories (*P* for trend < 0.001). Furthermore, in the multivariable-adjusted model, with BMI < 18.5 kg/m^2^, 24 ≤ BMI < 28 kg/m^2^, BMI ≥ 28 kg/m^2^ compared with 18.5 ≤ BMI < 24 kg/m^2^, the risk values of mortality were HRs: 1.33 (95% CI 1.18 to 1.49), 0.88 (0.83 to 0.93), and 0.90 (0.82 to 0.98), respectively, for NFG; 0.89 (0.55 to 1.46), 0.84 (0.71 to 0.99), and 0.88 (0.70 to 1.11), respectively, for IFG; and 1.42 (0.88 to 2.29), 0.75 (0.64 to 0.89), and 0.76 (0.62 to 0.93), respectively, for T2DM (Table 2).

### Restricted cubic spline analyses

**Fig. 2:**
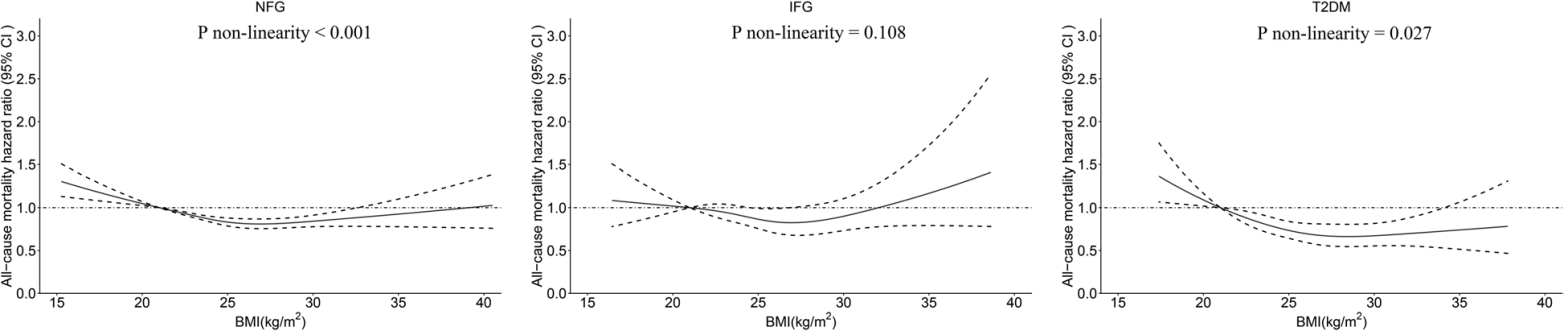
BMI and risk of all-cause mortality with NFG (**A**), IFG (**B**) and T2DM (**C**). Data are HRs (black line) and 95% CIs (gray shadow) from Cox regression analysis with restricted cubic splines, with BMI 21 kg/m^2^ as the reference. Multifactorial adjustment was for age, sex, smoking, alcohol consumption, physical activity, history of CHD, COPD, cancer and stroke, SBP and TC at baseline

We used restricted cubic spline analyses to examine the association between BMI on a continuous scale and all‐cause mortality after adjustment for age, sex, smoking, alcohol consumption, physical activity, history of CHD, COPD, cancer and stroke, SBP and TC at baseline, as shown in Fig. 2. For NFG and T2DM, curvilinear associations between BMI (as a continuous variable) and all-cause mortality were found (*P* overall < 0.001 and *P* overall < 0.001, respectively; *P* nonlinearity < 0.001 and *P* nonlinearity = 0.027, respectively; Fig. 2). For IFG, no significantly association between BMI and all-cause mortality was observed (*P* overall = 0.170; Fig. 2). With each SD (3.5 kg/m^2^) increase in BMI for T2DM, the risk of all-cause mortality was reduced by 14% (HR 0.86, 95% CI 0.80–0.93).

### Subgroup analyses

**Fig. 3:**
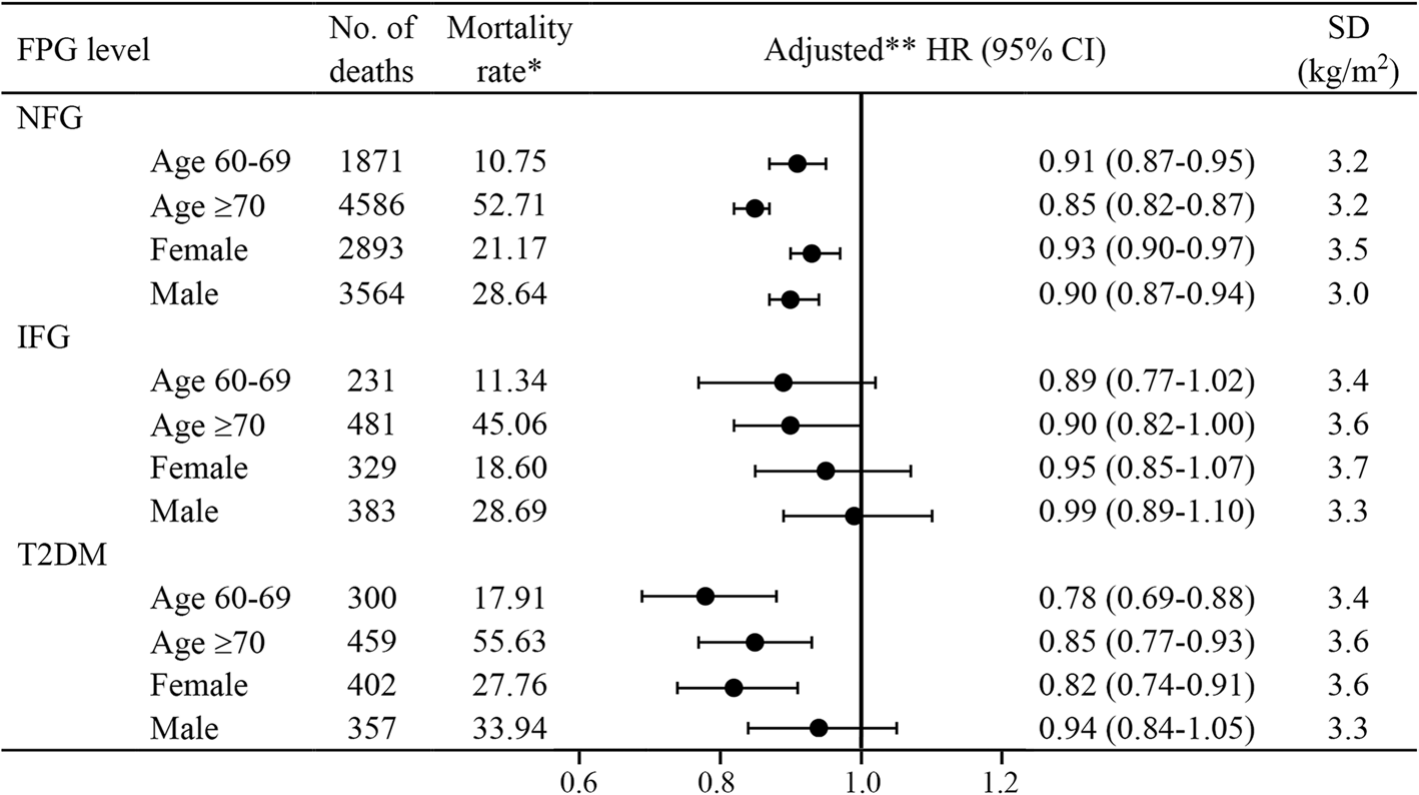
All-cause mortality for BMI per SD increase of glycemic status stratified by age and sex. One asterisk Per 1000 person-years; Two asterisks adjusted for sex, smoking, alcohol consumption, physical activity, history of CHD, COPD, cancer and stroke, SBP and TC at baseline when stratified by age, adjusted for age, smoking, alcohol consumption, physical activity, history of CHD, COPD, cancer and stroke, SBP and TC at baseline when stratified by sex

The analysis of all-cause mortality for BMI per SD change stratified by age and sex is shown in Fig. 3. The absolute mortality rate was higher for the ≥ 70 years age group than the < 70 years age group in each glycemic status. We also found higher absolute mortality rates among males in the three groups. In age and sex subgroups of NFG and T2DM, the relative risk of all-cause mortality decreased significantly with changes in BMI per SD. In the IFG group, the risk of all-cause mortality decreased in all subgroups as BMI increased, but not significantly except the ≥ 70 years age group. The results of the subgroup analysis yielded similar findings as our main results: The risk of all-cause mortality decreased with increasing BMI (per SD) in the NFG and T2DM groups.

### Sensitivity analyses

Finally, the findings of the sensitivity analyses were similar to our main results: Compared with 18.5 ≤ BMI < 24 kg/m^2^ (the reference range), low BMI significantly increased the risk of mortality among the NFG group (Table 3). With increasing BMI, the risk of mortality was reduced by varying degrees among the NFG and T2DM groups (Table 3).

**Table 3:** Sensitivity analysis for each FPG level: association between BMI and risk of all-cause mortality

BMI (kg/m^2^)	NFG			IFG			T2DM		
	Deaths (n)	Death rate^a^	aHR (95%CI)	Deaths (n)	Death rate^a^	aHR (95%CI)	Deaths (n)	Death rate^a^	aHR (95%CI)
Excluding deaths within 2 years of follow-up									
BMI < 18.5	258	44.52	1.33 (1.17–1.51)	14	30.93	0.83 (0.49–1.43)	15	68.57	1.37 (0.82–2.32)
18.5 ≤ BMI < 24	3404	26.28	1.00 (reference)	329	26.43	1.00 (reference)	315	35.15	1.00 (reference)
24 ≤ BMI < 28	1640	18.57	0.88 (0.83–0.93)	212	18.07	0.85 (0.71–1.01)	232	23.54	0.77 (0.65–0.91)
BMI ≥ 28	507	17.35	0.89 (0.81–0.98)	93	17.21	0.91 (0.72–1.15)	117	23.54	0.77 (0.62–0.95)
Excluding deaths within 2 years of follow-up and smokers									
BMI < 18.5	230	45.00	1.34 (1.18–1.54)	13	31.49	0.82 (0.47–1.43)	14	74.07	1.44 (0.84–2.48)
18.5 ≤ BMI < 24	2961	26.66	1.00 (reference)	294	26.54	1.00 (reference)	280	35.16	1.00 (reference)
24 ≤ BMI < 28	1431	18.69	0.88 (0.82–0.94)	181	17.64	0.84 (0.70–1.02)	205	23.78	0.81 (0.67–0.97)
BMI ≥ 28	437	16.93	0.89 (0.80–0.98)	85	17.57	0.93 (0.73–1.19)	97	22.05	0.72 (0.57–0.91)
Excluding deaths within 2 years of follow-up, smokers, and participants with history of CHD, COPD, cancer and stroke at baseline									
BMI < 18.5	175	44.01	1.37 (1.18–1.60)	10	29.38	0.66 (0.35–1.25)	11	80.44	1.68 (0.91–3.10)
18.5 ≤ BMI < 24	2370	26.37	1.00 (reference)	234	27.08	1.00 (reference)	211	34.58	1.00 (reference)
24 ≤ BMI < 28	1110	18.56	0.87 (0.81–0.94)	140	18.19	0.83 (0.67–1.03)	152	23.62	0.81 (0.65–1.00)
BMI ≥ 28	328	17.47	0.90 (0.80–1.01)	66	19.40	0.92 (0.70–1.22)	61	19.82	0.63 (0.47–0.84)

^a^Per 1000 person-years. HRs and 95% CIs were calculated using Cox hazards models after adjustment for age, sex, smoking, alcohol consumption, physical activity, history of CHD, COPD, cancer and stroke, SBP and TC at baseline

## Discussion

In this study of 59,874 participants from the elderly population, 10,817 (18.1%) had hyperglycemia (IFG and T2DM), highlighting a high prevalence of hyperglycemia in China’s county-level urban elderly population. There were curvilinear-shaped associations between BMI and mortality in the NFG and T2DM groups and no significant association in the IFG group. For people with NFG, lower BMI increased the risk of mortality. For people with NFG and patients with T2DM, compared with 18.5 ≤ BMI < 24 kg/m^2^, higher BMI had a protective effect on mortality risk. Meanwhile, the current study also found that there was almost no association between BMI categories and risk of mortality in the IFG group. These results were similarly maintained after sensitivity analyses, which suggests an obesity paradox, in which patients with an elevated BMI have a lower mortality rate than the normal-weight group. The BMI categories with the lowest risks in this study were overweight (24 ≤ BMI < 28 kg/m^2^) and/or obesity (≥ 28 kg/m^2^), whereas underweight (< 18.5 kg/m^2^) had the highest risks. In addition, we found a higher absolute mortality rate among males, but the relative magnitude of the effect on mortality risk was similar among both females and males. These findings indicated that there may be different associations between BMI and mortality among Chinese people with different glycemic statuses.

Our findings that overweight and obesity were protective factors for all-cause mortality in older adults with T2DM are consistent with previous studies on older adults [10, 21, 22]. The study of *Hui Liu* et al. observed that for patients with T2DM aged ≥ 60 years, the overweight and obesity groups were both protective against all-cause mortality compared with the normal weight group [10]. Similar results were observed by *Jihong Ma* et al. [21], and a relatively large prospective cohort including 3186 elderly patients confirmed the obesity paradox for the 6-year and 9-year all-cause mortalities seen in China [22].

Information on the association between BMI and mortality in older Asian diabetic patients (Singapore [23], Korea [24], China [10, 22]) is limited. A Singapore study found that being overweight (23.0–27.5 kg/m^2^), but not obese (≥ 27.5 kg/m^2^), was associated with a reduced risk of death among T2DM patients aged 65 or older. However, the follow-up time was relatively short, with a median follow-up period of 2.9 years [23]. A dose–response meta-analysis in South Korea reported that with a BMI nadir of 28–30 kg/m^2^, the risk of all-cause mortality displayed a U-shaped increase with age of 65 years or older and was not a source of heterogeneity among studies [24]. However, some studies in the meta-analysis used self-reported BMI, while others used standardized measures. In China, recent research from the Kailuan Study showed a U-shaped association between BMI and 7-year all-cause mortality in 11,449 adults with T2DM [10]. However, this study should also be interpreted with caution due to the presence of gender imbalance (male: 83.1%), a biased population (occupational population) and insufficient explanation of potential confounding factors. Besides, Korean Metabolic Risk Factor Study had demonstrated that U-curve relationships existed regardless of diabetes status [25]. The reasons why the result of these studies differ from previous research may lie in differences in study populations, follow-up periods, related confounding factors included and data definitions used.

This study found that males had a higher absolute mortality rate than females in the entire older population, which was consistent with previous findings [13, 26]. However, the observed magnitude of the mortality effect size was similar for both females and males. Potential explanations for the phenomenon include women’s greater use of medical services, physician diagnostic patterns, as well as the idea that women are more willing to acknowledge and report illness [26]. Sex differences in illness persist: Women appear to have higher rates of conditions that rarely cause death, whereas men tend to have more fatal conditions [26, 27]. Meanwhile, men had higher mortality risk than women because men were exposed to more risk factors than women, such as smoking and alcohol consumption.

There are several other possible explanations for the obesity paradox. BMI is only a rough measure of obesity, and a low BMI may reflect sarcopenia (loss of muscle mass), which in turn may reflect factors such as catabolic status, malnutrition, comorbidities or reduced physical activity [28–30]. Low BMI is also predictive of increased mortality; this could potentially be mediated through known associations with weight loss, chronic diseases, frailty, and cachexia [6, 7]. In older adults with low BMIs, frequent falls can lead to multiple illnesses, although these events were not recorded in our database. Excess weight may result in metabolic reserves that protect against adverse outcomes. Another possible explanation is that obese patients are more likely to be screened for diabetes, leading to an earlier diagnosis. Being overweight may provide a metabolic reserve in older patients, protecting against frailty, malnutrition, and osteoporosis [31, 32]. Although our results confirm the obesity paradox in older patients with T2DM, the harmful impact of obesity on health cannot be ruled out. Obesity is a risk factor for hypertension, diabetes, myocardial infarction, stroke and other diseases [33–35]. Obesity is also associated with an increased risk of more than 10 cancers, such as uterine cancer, cervical cancer and colorectal cancer [36]. Therefore, weight loss should be emphasized as a primary preventive measure of cardiovascular and cerebrovascular events. However, to avoid an increased risk of death, weight loss should not be overemphasized when certain complications occur.

The main strengths of this prospective cohort study include the large sample size (59,874 adults) composed of participants aged 60 and over who had comorbidities (CHD, COPD, cancer or stroke), or BMI < 18.5 kg/m^2^, as well as the median of 5.96 years of follow-up, with these attributes, our study was an accurate representation of the older population. The BMI cutoff points used in our study were also the most suitable for the Chinese population, allowing for evaluation of the BMI–mortality association by diabetes status and multivariate analyses to deal with potential confounders, including age, sex, smoking, alcohol consumption, physical activity, history of CHD, COPD, cancer and stroke, SBP and TC at baseline. The results remained unchanged in the sensitivity analysis. Furthermore, we established nonlinear relationships between BMI and risk for all-cause mortality via the natural spline function test among the NFG and T2DM groups, and inverse J curves were identified. Therefore, the findings of this study were credible. The current study has some limitations. First, prevalent diabetes and newly diagnosed diabetes may influence the BMI–mortality association because diabetes treatment often leads to weight loss, or because long-standing diabetes may lead to complications and death. However, data on the duration of diabetes are lacking. Second, the limited investigation time, with a median follow-up of 5.96 years, may not be sufficient for all end points of interest to occur. Lastly, we had no information on socioeconomic and nutritional status, medical history of falls, or social support, which might be particularly relevant to these older adults. More studies investigating all-cause mortality among older Chinese people with different BMI levels by glycemic status is needed to verify these results and enhance the accuracy of our study.

## Conclusion

In this 9‐year cohort study of older Chinese adults, curvilinear associations between BMI and all‐cause mortality were demonstrated for glycemic status in the NFG and T2DM groups, and no significantly association was demonstrated in the IFG group. For Chinese patients with diabetes, both overweight and obesity significantly decreased the risk of mortality compared with normal weight, while underweight increased the risk of mortality, being not significantly. Future studies that aim to determine the relationship between BMI and all-cause mortality in older adults need to be cognizant of the effects of fasting glucose levels.

## Data Availability

The datasets generated and/or analysed during the current study are not publicly available due confidentiality requirements but are available from the corresponding author on reasonable request.

## Abbreviations

BMI: Body mass index
NFG: Normal fasting glucose
IFG: Impaired fasting glucose
T2DM: Type 2 diabetes mellitus
HRs: Hazard ratios
CIs: Confidence intervals
FPG: Fasting plasma glucose
DALYs: Disability-adjusted life years
CVD: Cardiovascular disease
TC: Total cholesterol
TG: Triglyceride
CHD: Coronary heart disease
COPD: Chronic obstructive pulmonary disease
SBP: Systolic blood pressure
IQR: Interquartile range
